# Phenotypic heterogeneity in *IGHV*-mutated CLL patients has prognostic impact and identifies a subset with increased sensitivity to BTK and PI3Kδ inhibition

**DOI:** 10.1038/leu.2014.308

**Published:** 2014-11-18

**Authors:** C Pepper, A G S Buggins, C H Jones, E J Walsby, F Forconi, G Pratt, S Devereux, F K Stevenson, C Fegan

**Affiliations:** 1 Cardiff CLL Research Group, Institute of Cancer & Genetics, School of Medicine, Cardiff University, Cardiff, UK; 2 Department of Haematology, King's College London, London, UK; 3 Cancer Sciences Unit, CRUK Clinical Centre, University of Southampton, Southampton, UK; 4 CRUK Institute for Cancer Studies, University of Birmingham, Birmingham, UK

The majority of chronic lymphocytic leukemia (CLL) patients are diagnosed with early-stage disease but the currently used prognostic tools appear to be less informative in this group of patients.^1^ This is especially problematic for patients with mutated immunoglobulin genes (M-CLL) as they have a more diverse clinical course when compared with patients with unmutated immunoglobulin genes (U-CLL).^1, 2, 3, 4^ Given the emergence of promising targeted, less toxic, therapeutics in CLL,^5, 6^ there is an increased need to identify patients who might benefit from early treatment with these new agents.

Chemokine receptors expressed on CLL cells are thought to regulate the trafficking of the leukemic cells between blood and lymphoid tissues.^5^ Logically, the tendency of CLL cells to return to tissue sites where they are cytoprotected and are driven to proliferate contributes to clinical aggressiveness. It is therefore possible that these receptors represent promising prognostic markers and potentially useful therapeutic targets. We previously reported that one such chemokine receptor, CXCR4, is associated with poor clinical outcome in an unselected cohort of CLL patients.^7^ Here we specifically focused our attention on M-CLL samples to ascertain the impact of CXCR4 expression in this clinically heterogeneous subset. The cohort was made up of 60 M-CLL patients from the original study and 64 additional M-CLL patients. The patient characteristics are given in Supplementary Table 1. CXCR4 expression was determined using the three-color flow cytometric assay described previously.^7^ We used the median expression as a binary classifier based on receiver operating characteristic analysis. Of the 124 M-CLL samples analyzed, 50 (40.4%) were classified as CXCR4^hi^ and 74 (59.6%) as CXCR4^lo^; M-CLL patients showed much more heterogeneity in CXCR4 expression than U-CLL (Figure 1a). Importantly, elevated CXCR4 expression in M-CLL was a strong determinant of reduced overall survival (Figure 1b; hazard ratio (HR)=3.5). As M-CLL patients utilizing *IGHV*3-21 genes have been shown to have an inferior clinical outcome,^8, 9^ we asked whether the CXCR4 expression was significantly different in the *IGHV*3-21 subset. We found no significant increase in CXCR4 expression in *IGHV*3-21 samples when compared with samples utilizing other *IGHV* gene segments (Supplementary Figure 1; *P*=0.92). Similarly, we found no association between CXCR4 expression and the high-risk cytogenetic risk groups (Supplementary Figure 1; *P*=0.41).

We recently showed a strong association between CXCR4 expression and CD49d in an unselected cohort of U-CLL and M-CLL patients.^7^ We therefore examined the expression of CD49d in this M-CLL subset using the same flow cytometric methodology and determined its prognostic relevance. In concordance with our CXCR4 data, there was heterogeneous expression of CD49d with 60 (47.4%) CD49d^hi^ and 64(52.6%) CD49d^lo^. Again this heterogeneity was in marked contrast to U-CLL cases (Figure 1c). Furthermore, CD49d^hi^ patients (>30% positive) had a significantly worse clinical outcome than CD49d^lo^ patients (Figure 1d; HR=3.4) reinforcing the credentials of CD49d as a prognostic marker in the M-CLL subset.^10^ When assessed as continuous variables, CXCR4 and CD49d were strongly correlated (Figure 1e; *P*<0.0001). Using categorical cutoffs to define the cohort, the majority of M-CLL cases showed concordant expression for CXCR4 and CD49d: CXCR4^hi^/CD49d^hi^ or CXCR4^lo^/CD49d^lo^. However, 27% of the subset was discordant for these markers (Supplementary Figure 2). The Kaplan–Meier curve for the discordant cases bisected the CXCR4^hi^/CD49d^hi^ and CXCR4^lo^/CD49d^lo^ curves highlighting the prognostic importance of both CXCR4 and CD49d and suggesting that the combined assessment of CXCR4 and CD49d has clinical utility. This was supported by the observation that the combination of CXCR4 and CD49d was a more powerful prognostic tool than either marker alone for the concordant cases (Figure 1f; HR=5.2). It is worthy of note that CXCR4^hi^/CD49d^hi^ M-CLL cases had a similar clinical outcome when compared with U-CLL cases (Supplementary Figure 3).

We went on to establish the functional significance of CXCR4 and CD49d expression in M-CLL. CXCR4^hi^/CD49d^hi^ samples showed significantly increased migration in response to CXCL12 in transwell experiments (Figure 2a; *P*=0.012), which could be inhibited by both plerixafor (CXCR4 antagonist) and natalizumab (anti-CD49d antibody) even in the presence of CXCL12 (Figure 2b). We observed a significant reduction in CXCR4 expression when CLL cells were incubated with CXCL12 presumably owing to the internalization of the receptor (Supplementary Figure 4a). No change in CD49d expression was seen under these conditions (Supplementary Figure 4b). There is now a growing appreciation that the propensity of CLL cells to home to lymphoid tissues contributes to the clinical course of CLL.^11, 12^ We recently showed that CXCR4^hi^ and CD49d^hi^ samples have a higher propensity to undergo transendothelial migration in a dynamic *in vitro* model of the peripheral vasculature of CLL suggesting that these molecules contribute to this process.^12^ The importance of CLL cell migration is perhaps best exemplified by the remarkable clinical effect of the BTK and PI3Kδ inhibitors ibrutinib and idelalisib.^13^ Both drugs promote a redistribution of CLL cells into the peripheral vasculature from lymphoid tissues characterized by a rapid increase in peripheral lymphocytosis. These cells appear then to be trapped in the peripheral vasculature where, in the absence of prosurvival and proproliferative signals, they are potentially more susceptible to spontaneous apoptosis and chemotherapeutic agents. Although these drugs are currently being trialed in patients with progressive or advanced stage disease,^14, 15^ their side-effect profiles are modest and so it seems likely that they will be considered as frontline treatment options in early-stage asymptomatic patients. This being the case, it will be crucial to accurately identify those patients who might benefit from early treatment and equally those who may not require treatment at all. Intriguingly, a recent study indicated that M-CLL patients had an inferior response to ibrutinib when compared with U-CLL patients.^6^ Here we show that both drugs caused a significant reduction in CXCR4 expression (Figure 2c) and CD49d expression (Figure 2d). Furthermore, despite the enhanced migratory properties of CXCR4^hi^/CD49d^hi^ M-CLL samples ibrutinib and idelalisib induced a significantly greater percentage reduction in the migration of CXCR4^hi^/CD49d^hi^ M-CLL samples (mean migration 9.44±2.08% to 4.00±1.12%) when compared with CXCR4^lo^/CD49d^lo^ samples (mean migration 8.75±1.61% to 4.97±0.85% Figure 2e). In addition, CXCR4^hi^/CD49d^hi^ M-CLL samples were more susceptible to the apoptotic effects of ibrutinib and idelalisib (Figure 2f). These findings suggest that CXCR4^hi^/CD49d^hi^ M-CLL patients are more dependent on BTK and PI3Kδ signaling, and hence could particularly benefit from treatment with inhibitors of these kinases.

Taken together, our data provide evidence that CXCR4 and CD49d are important modulators of prognosis in M-CLL patients. Furthermore, the combination of CXCR4 and CD49d measurement provided even greater prognostic resolution for concordant M-CLL cases. This knowledge could facilitate enhanced flow cytometry-based risk stratification for this heterogeneous group of patients and identify those who might particularly benefit from BTK or PI3Kδ inhibitors or therapeutics designed to target the function of CXCR4 and CD49d. Similarly, it could delineate a substantial population of CXCR4^lo^/CD49d^lo^ M-CLL patients with very low risk of disease progression who could be monitored less frequently.

## Figures and Tables

**Figure 1:**
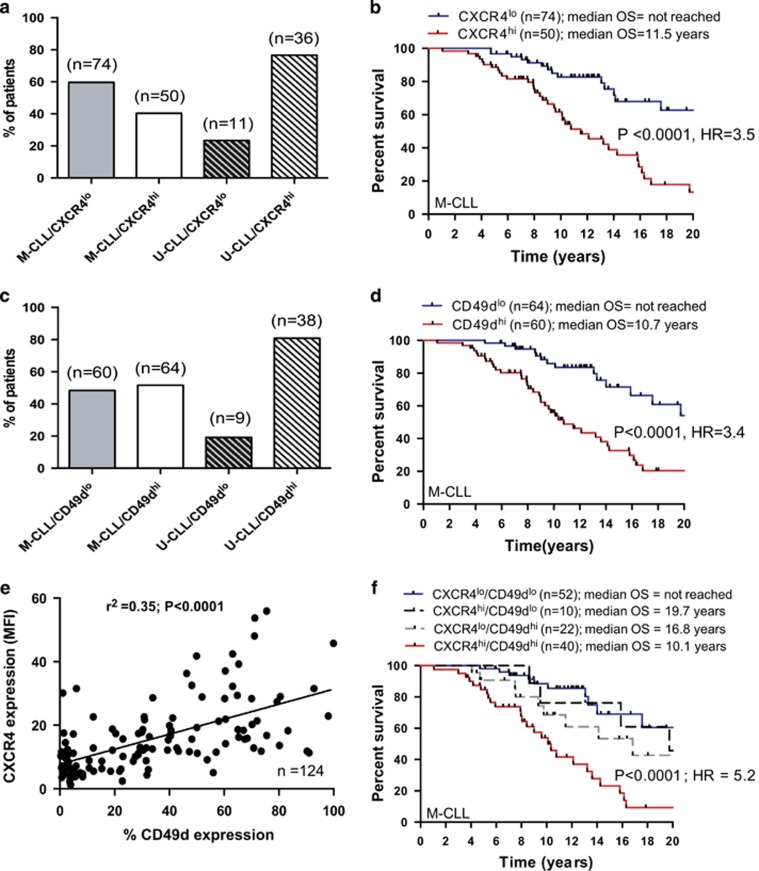
Differential expression and prognostic impact of CXCR4 and CD49d in M-CLL. (**a**) We analyzed the expression of CXCR4 in a cohort of 124 serially collected M-CLL samples and used the median expression as a binary classifier based on receiver operating characteristic analysis. M-CLL patients showed a much more heterogeneous expression of CXCR4 than U-CLL cases. (**b**) M-CLL patients with a CXCR4^hi^ phenotype had a significantly inferior outcome when compared with CXCR4^lo^ patients. (**c**) The cohort was then categorized into CD49d^lo^ (<30% expression) and CD49d^hi^ (⩾30% expression) subsets. Again M-CLL cases showed much more heterogeneity in the expression of CD49d than U-CLL cases. (**d**) M-CLL patients with a CD49d^hi^ phenotype had a significantly inferior outcome when compared with CD49d^lo^ patients. (**e**) When assessed as continuous variables, CXCR4 and CD49d were strongly correlated. Similarly, using categorical cutoffs to define the cohort, the majority of M-CLL cases showed concordant expression for CXCR4 and CD49d: CXCR4^hi^/CD49d^hi^ or CXCR4^lo^/CD49d^lo^. 27% of the cases were discordant for these markers. (f) The Kaplan–Meier curves for the discordant cases bisected the CXCR4^hi^/CD49d^hi^ and CXCR4^lo^/CD49d^lo^ curves highlighting the prognostic importance of both CXCR4 and CD49d and suggesting that the combined assessment of CXCR4 and CD49d has clinical utility.

**Figure 2:**
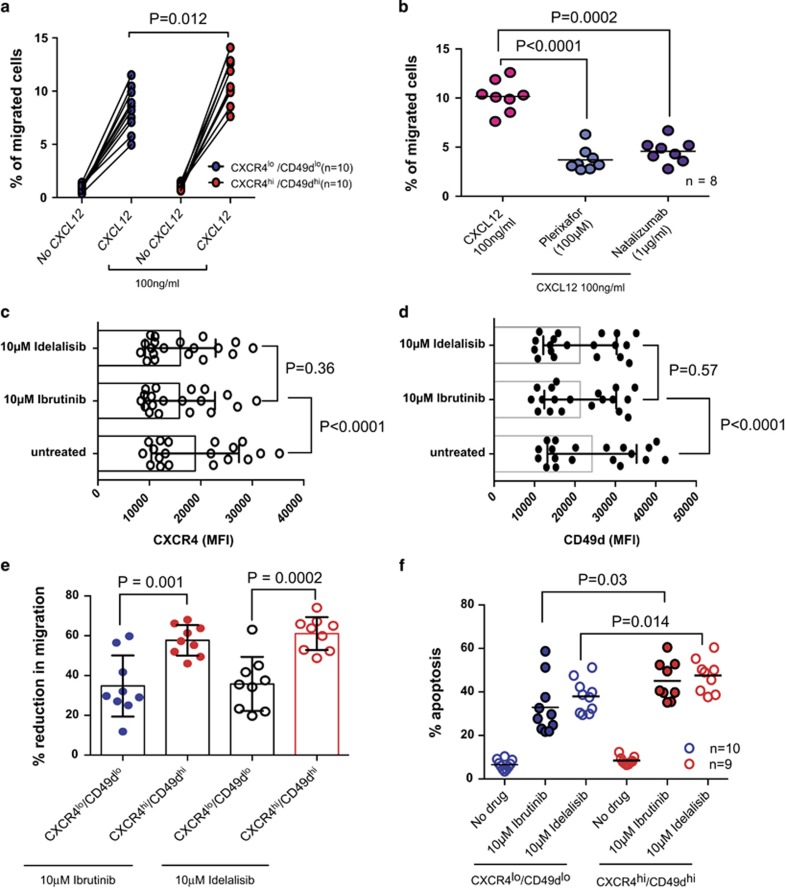
CXCR4^hi^/CD49d^hi^ M-CLL cases are functionally distinct from CXCR4^lo^/CD49d^lo^ M-CLL cases. *In vitro* migration assays were performed using 3.0-μm pore size transwell migration plates (Costar, Corning, NY, USA). A total of 10^6^ CLL cells in 500 μl of RPMI containing 10% fetal calf serum (FCS) were added to the upper chamber of the transwell insert. 100 ng/ml of CXCL12 was added to the lower wells. The plates were incubated for 4 h at 37 °C in 5% CO_2_. Controls without CXCL12 were maintained in each experiment to account for passive diffusion of cells (<1.5% in every case). (**a**) CXCR4^hi^/CD49d^hi^ samples (*n*=10) showed significantly increased migration in response to CXCL12 when compared with CXCR4^lo^/CD49d^lo^ samples (*n*=10). (**b**) Addition of plerixafor (100 μM) or natalizumab (1 μg/ml) to the upper chamber of the transwell insert significantly inhibited CLL cell migration despite the presence of a CXCL12 chemokine gradient (*n*=8). Exposure to ibrutinib or idelalisib for 24 h resulted in a significant reduction in (**c**) CXCR4 expression and (**d**) Cd49d expression in the viable CD19^+^/CD19^+^ CLL cells. (**e**) Although CXCR4 ^hi^/CD49d^hi^ samples were inherently more migratory than CXCR4^lo^/CD49d^lo^ samples, incubation with ibrutinib (10 μM) and idelalisib (10 μM) for 4 h induced a significantly larger percentage reduction in migration in CXCR4^hi^/CD49d^hi^ samples. (**f**) CLL cells derived from M-CLL patients were maintained *in vitro* in RPMI supplemented with 10% FCS and 5 ng/ml interleukin-4 in the presence or absence of 10 μM ibrutinib. After 48 h apoptosis was assessed using Annexin V and propidium iodide labeling. CXCR4^hi^/CD49d^hi^ samples (*n*=9) showed significantly increased apoptotic cell death in response to ibrutinib and idelalsib when compared with CXCR4^lo^/CD49d^lo^ samples (*n*=10). All paired and unpaired observations were analyzed using the Student's *t*-test after confirming that the data were Gaussian or a Gaussian approximation using the omnibus K2 test.
