# Supplementary material for: Phenotypic heterogeneity in IGHV-mutated CLL patients has prognostic impact and identifies a subset with increased sensitivity to BTK and PI3Kδ inhibition
Source: Leukemia. 2014 Nov 18;29(3):744–7. doi: 10.1038/leu.2014.308 (PMC4360209; doi:10.1038/leu.2014.308)
Supplement: Supplementary Table 1 [file leu2014308x1.doc]

**Supplementary Table 1. Clinical characteristics of the 124 CLL patient cohort.**

| Factor | Subset | Number |
| --- | --- | --- |
| Median Age | | 65 years |
| Range | | 34 - 93 years |
| Median Follow up | | 9.8 years |
| Binet stage at diagnosis | A | 103 |
|  | B | 15 |
|  | C | 6 |
| Required treatment | Treated | 32 |
|  | Untreated | 92 |
| CD38 | <20% | 76 |
|  | ≥20% | 48 |
| Genetics | 11q^-^ / 17p^-^ | 7 |
|  | N / O | 80 |
|  | Not Determined | 37 |
| *IGHV* Status | M-CLL | 124 |
| ZAP-70 | <20% | 82 |
|  | ≥20% | 42 |
| CD49d | <30% | 69 |
|  | ≥30% | 56 |

11q^-^ and 17p^-^: any FISH or karyotypic abnormality involving 11q or 17p

N: No detectable cytogenetic aberration by FISH; O: Other cytogenetic abnormality (excluding 11q^-^ or 17p^-^)

*IGHV* status: M-CLL: <98% sequence homology with the closest germline sequence
