# Supplementary figures and images for: Phenotypic heterogeneity in IGHV-mutated CLL patients has prognostic impact and identifies a subset with increased sensitivity to BTK and PI3Kδ inhibition
Source: Leukemia. 2014 Nov 18;29(3):744–7. doi: 10.1038/leu.2014.308 (PMC4360209; doi:10.1038/leu.2014.308)

Supplementary Figure 1

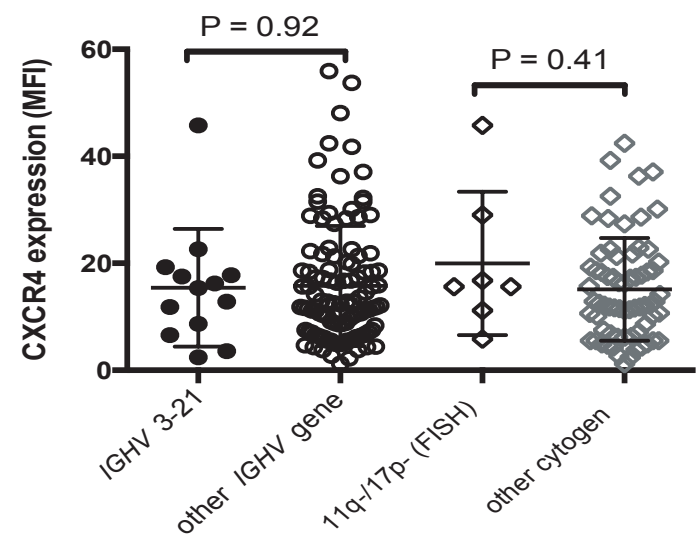

Supplement: Supplementary Figure 1 [file leu2014308x2.pdf]

Supplementary Figure 2

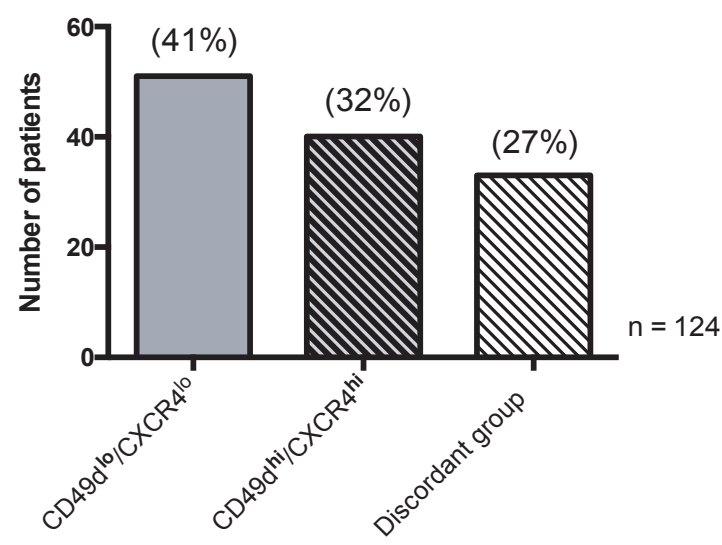

Supplement: Supplementary Figure 2 [file leu2014308x3.pdf]

**Supplementary Figure 3**

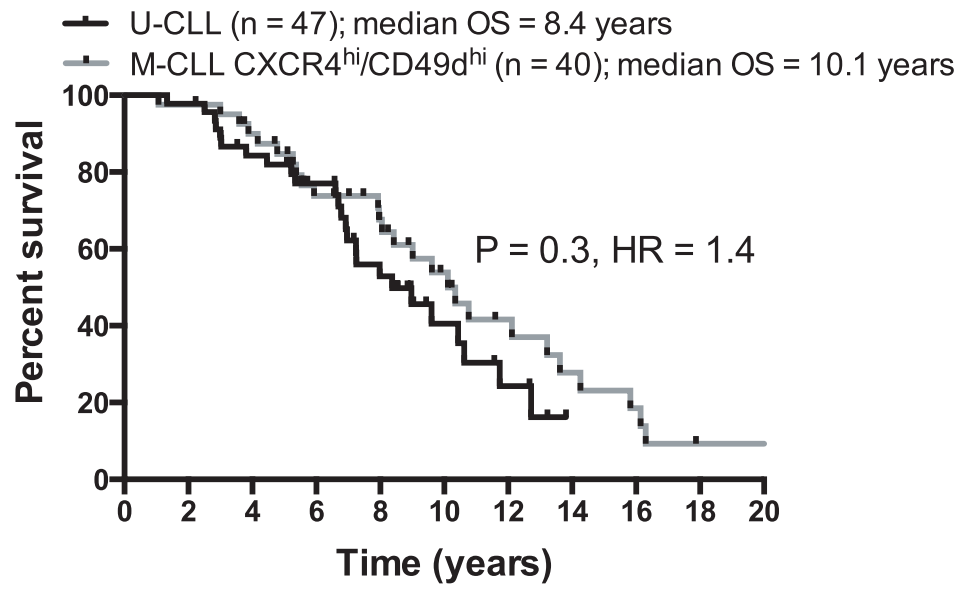

Supplement: Supplementary Figure 3 [file leu2014308x4.pdf]

Supplementary Figure 4

a

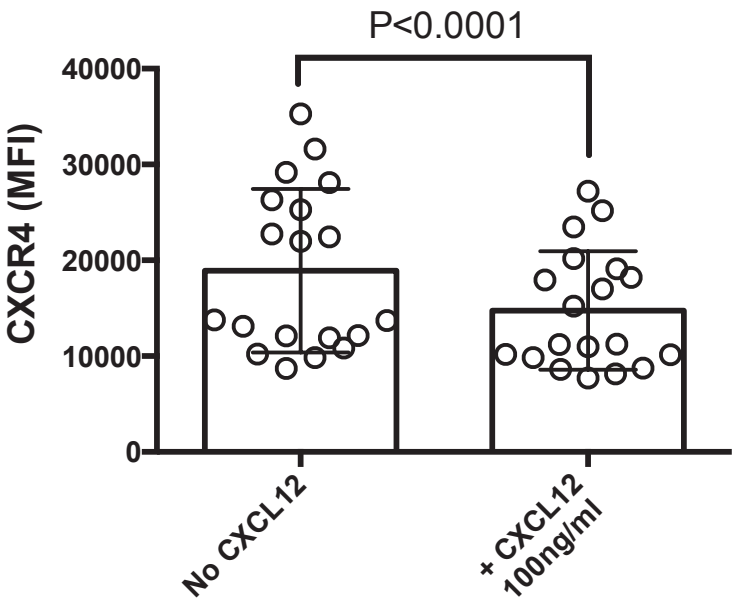

b

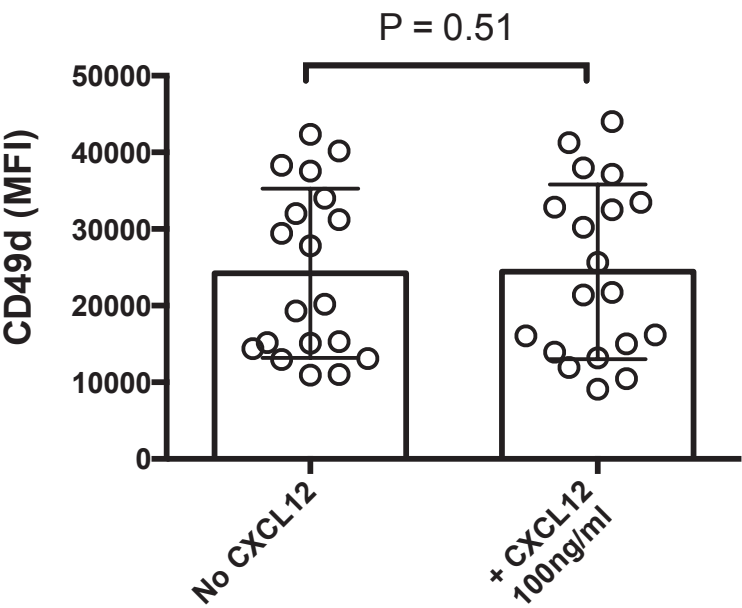

Supplement: Supplementary Figure 4 [file leu2014308x5.pdf]
