# Supplementary material for: Phenotypic heterogeneity in IGHV-mutated CLL patients has prognostic impact and identifies a subset with increased sensitivity to BTK and PI3Kδ inhibition
Source: Leukemia. 2014 Nov 18;29(3):744–7. doi: 10.1038/leu.2014.308 (PMC4360209; doi:10.1038/leu.2014.308)
Supplement: Supplementary Figure Legends [file leu2014308x6.doc]

**Supplementary Figure legends**

**Supplementary Figure 1**

The inferior prognosis associated with elevated CXCR4 expression in the M-CLL cohort could not be explained by differential *IGHV*3-21 gene usage or a preponderance of high-risk cytogenetic lesions in the CXCR4^hi^ patients.

**Supplementary Figure 2**

Using categorical cut-offs to define the cohort, the majority of M-CLL cases showed concordant expression for CXCR4 and CD49d: CXCR4^hi^/CD49d^hi^ or CXCR4^lo^/CD49d^lo^. However, 27% of the subset was discordant for these markers.

**Supplementary Figure 3**

Assessment of the CXCR4 and CD49d concordant and discordant M-CLL cases in terms of overall survival showed that the CXCR4^hi^/CD49d^hi^ patients had a significantly worse outcome than discordant cases and those with CXCR4^lo^/CD49d^lo^ phenotype. Furthermore, CXCR4^hi^/CD49d^hi^ M-CLL patients showed similar survival to U-CLL cases.

**Supplementary Figure 4**

The addition of CXCL12 (100ng/ml) to CLL cell cultures resulted in **(a)** a significant reduction in CXCR4 expression on the surface of CD5+/CD19+ CLL cells. **(b)** In contrast, CXCL12 had no effect on CD49d expression.
